# Anatomical Variations in the Formation of the Sural Nerve: A Pilot Study in a Sample of Lithuanian Cadavers

**DOI:** 10.3390/medicina61040671

**Published:** 2025-04-05

**Authors:** Artur Airapetian, Benedikt Bachmetjev, Andrej Suchomlinov

**Affiliations:** 1 Faculty of Medicine, Vilnius University, M. K. Ciurlionio 21, LT-03101 Vilnius, Lithuania; benedikt.bachmetjev@mf.stud.vu.lt; 2 Department of Anatomy, Histology and Anthropology, Institute of Biomedical Sciences, Faculty of Medicine, Vilnius University, LT-03101 Vilnius, Lithuania; andrej.suchomlinov@mf.vu.lt; 3 Division of Anatomy, Department of Surgery, School of Medicine, University of California San Diego, La Jolla, CA 92093, USA

**Keywords:** sural nerve, anatomical variations, cadaveric study

## Abstract

*Background and Objectives*: The sural nerve (SN) is a pure sensory nerve that supplies the lateral aspect of the ankle and foot. Its anatomical variability has been extensively documented, with multiple classifications describing its different formation patterns. The SN is commonly used for nerve grafting and is a critical structure in lower-limb surgeries. Due to its superficial course, it is vulnerable to iatrogenic injuries, particularly in procedures involving the Achilles tendon. The presence of anatomical variations in SN formation and trajectory has significant implications for surgical planning, diagnostics, and nerve conduction studies. Understanding these formation variations is essential to minimize surgical complications and optimize clinical outcomes. *Materials and Methods*: A pilot cross-sectional cadaveric study was conducted on nine formalin-fixed adult cadavers at the Department of Anatomy, Histology, and Anthropology, Vilnius University Faculty of Medicine, Lithuania. Standard dissection techniques were employed to examine the formation and trajectory of the SN. Morphometric parameters, including nerve diameter and length, were measured using an RS PTO Digital Caliper with 0.01 mm precision. Variations in SN formation were classified according to the system proposed by P.K. Ramakrishnan et al. Statistical analyses were performed using SPSS 26.0 and RStudio, with a significance threshold set at *p* ≤ 0.05. *Results*: The most prevalent SN formation variation observed in the Lithuanian cadaveric sample was Type 3, which was found in 8 out of 18 limbs (44.4%), while Type 6 was not identified. Additionally, a symmetric formation was observed bilaterally in 5 out of the 9 cadavers (55.6%). The SN was significantly thicker in two-contributor formations (3.17 mm) compared to single-contributor formations (1.93 mm, *p* = 0.001). The SN was also significantly longer in two-contributor formations (25.80 cm) than in single-contributor formations (18.96 cm, *p* = 0.016). No significant differences in SN morphology were found between left and right lower limbs. *Conclusions*: This study highlights the substantial anatomical variability of the SN in the Lithuanian population. The findings suggest a correlation between SN diameter and formation type, which may have clinical implications for nerve grafting and surgical planning. The predominance of Type 3 formation and the observed symmetry rate provide valuable anatomical insights for lower limb surgeries. Further large-scale studies are necessary to establish population-specific SN variations and their relevance in clinical practice.

## 1. Introduction

The sural nerve (SN) is a significant pure sensory cutaneous nerve that innervates the base of the fifth metatarsal, as well as the lateral ankle and foot [1]. It is formed by contributions from the tibial nerve (TN) and the common peroneal nerve (CPN), with several distinct variations in its formation reported in the literature [2,3,4]. The peroneal communicating branch (PCB) of the common peroneal nerve and the medial sural cutaneous nerve (MSCN), a branch of the tibial nerve, are two major components that frequently unite to form the SN [5,6,7,8,9]. However, in some cases, the SN may arise directly from the MSCN, the lateral sural cutaneous nerve (LSCN), or as a fusion of the MSCN and LSCN, branching from the common peroneal nerve [7]. The sural nerve is typically formed within the anatomical region extending from the popliteal fossa to the level of the ankle joint [10]. It runs along the lateral border of the Achilles tendon (TC), near the small saphenous vein (SSV), and between the heads of the gastrocnemius muscle [11,12]. As it courses posteriorly and inferiorly toward the lateral malleolus, its superficial location becomes particularly relevant for surgical procedures. Due to its predictable anatomical landmarks and accessibility, the sural nerve is often harvested near the lateral malleolus for nerve grafting. Despite its relatively consistent trajectory, the sural nerve exhibits considerable anatomical variability in its origin and formation. Multiple classification systems have identified at least four distinct formation patterns, which influence its course, branching patterns, and potential clinical implications. These variations must be carefully considered in both surgical and diagnostic procedures to minimize complications and optimize patient outcomes [13,14,15].

Sural nerve pathology often presents with nonspecific symptoms, such as hypoesthesia and neuropathic pain, significantly impacting the quality of life [16,17]. Although high-frequency ultrasound is commonly used for SN imaging, the nerve’s small size and complex fascicular structure pose challenges. Due to the anatomical variability in the sural nerve (SN) at its origin, locating it distally and tracing it proximally is often a more effective approach for identification [18]. Magnetic resonance imaging (MRI) provides better visualization of the SN’s anatomical course, making it a valuable tool for surgical planning. However, MRI has limitations in clearly differentiating fascicular structures. In contrast, micro-CT, when combined with contrast agents, allows for high-resolution three-dimensional reconstruction, offering superior detail in fascicular organization. These advanced imaging techniques play a crucial role in early diagnosis, guiding treatment, and preventing iatrogenic injury during surgical procedures [19]. One of the surgical procedures frequently associated with SN injury involves minimally invasive Achilles tendon repair. The incidence of sural nerve paresthesia following surgical intervention for Achilles tendon repair varies significantly, ranging from 1.7% to 23% in percutaneous procedures. However, when all surgical techniques are considered, the overall occurrence of paresthesia can be as high as 60%. Postoperative sural nerve dysfunction may result from either traction or sharp injury, often occurring during the dissection of the Achilles tendon’s connective tissue (peritendineum), at the site of the stab incision, or during the creation of the canal through the lateral calcaneus. Notably, both open and percutaneous repair techniques carry a risk of sural nerve damage. However, percutaneous suturing is the primary cause of nerve entrapment in these cases, highlighting the need for careful surgical planning and technique selection to minimize the risk of complications [20].

The sural nerve is widely recognized by surgeons as a preferred site for harvesting autologous nerve grafts [21]. It is particularly advantageous for nerve grafting due to its considerable length, expandable nature, and optimal caliber for revascularization in interfascicular graft replacement [22]. These characteristics make the sural nerve an excellent choice for repairing nerve defects resulting from traumatic injuries. In clinical practice, the sural nerve is typically identified in relation to the small saphenous vein, which serves as a reliable anatomical landmark. However, due to the inherent variability in its formation, surgeons may need to assess both legs to determine the most suitable graft specimen. This ensures optimal graft selection and improves surgical outcomes. Sural nerve grafts are especially valuable in reconstructive procedures, playing a crucial role in restoring muscle tone in cases of facial nerve palsy. Their effectiveness in nerve repair highlights the importance of understanding sural nerve anatomy and its variations to enhance surgical precision and patient recovery [23].

Sural nerve biopsy is a valuable diagnostic tool for assessing peripheral neuropathies, particularly in identifying underlying inflammatory mechanisms [24]. While often unnecessary when neuropathy can be diagnosed through clinical and laboratory tests, it becomes essential in cases of suspected vasculitis or unexplained peripheral neuropathy [25]. The sural nerve is an ideal candidate for biopsy due to its superficial location, predictable sensory distribution, and purely sensory function, which minimize the risk of motor deficits and ulceration [26]. The procedure is performed with the patient in a supine position under general anesthesia. A small incision is made approximately 10 cm below the popliteal fossa, and a 2–3 cm segment of the nerve is excised. The proximal portion of the nerve is then implanted into the gastrocnemius muscle to prevent the formation of painful neuromas [27]. A histopathological analysis of the excised nerve segment focuses on the examination of axons, myelin, and diagnostic lesions, such as amyloid deposits, sarcoid tubercles, and vasculitis. Inflammatory neuropathies are often characterized by pathological changes in endothelial cells and pericytes, which play a crucial role in the diagnostic process. Sural nerve biopsy is particularly important in cases of vascular neuropathy, where vasculitis leads to damage to the vasa nervorum, resulting in ischemia and symptoms such as pain, weakness, and sensory deficits [28,29,30,31]. Vasculitic neuropathy is typically marked by fibrinoid necrosis, asymmetric axonal loss, and perivascular microfasciculation [32].

In chronic inflammatory demyelinating polyneuropathy (CIDP), biopsy is generally reserved for atypical cases or when differentiation from vasculitis or amyloid deposition is necessary. CIDP typically presents as either symmetric or multifocal neuropathy, characterized by progressive weakness and sensory deficits. Histopathological findings in CIDP include axonal loss, inflammatory infiltrates, onion bulb formations, and segmental demyelination [32]. In summary, the sural nerve exhibits considerable anatomical variation, not only in its formation but also in its trajectory and branching patterns. These variations have significant clinical implications, particularly in surgical procedures, diagnostic imaging, and nerve grafting. Research has demonstrated that the frequency and types of sural nerve formation variations differ across populations from various geographical regions and ethnic backgrounds, likely due to genetic and developmental factors [33]. Understanding these anatomical differences is crucial for tailoring surgical approaches, minimizing the risk of iatrogenic injuries, and improving diagnostic accuracy. In nerve grafting procedures, recognizing regional anatomical variability ensures optimal donor site selection and reduces the likelihood of complications. Additionally, population-specific anatomical studies play a vital role in refining nerve conduction studies and enhancing the interpretation of biopsy results in neuropathy cases. Therefore, comprehensive anatomical research across diverse populations is essential for advancing clinical outcomes, improving patient safety, and optimizing both surgical and diagnostic strategies [33].

## 2. Materials and Methods

### 2.1. Study Design

A pilot cross-sectional cadaveric study was conducted to identify and analyze the variations of the sural nerve formation within the Lithuanian population. This pilot study provides foundational data on sural nerve formations variations in this population, focusing on anatomical and morphometric characteristics. The study systematically assessed the nerve’s course of variation, including critical parameters such as length, width, and the point of sural nerve formation.

This research not only recorded anatomical distinctions but also aimed to establish a baseline for understanding the population-specific patterns that might influence surgical interventions, diagnostic accuracy, and the development of nerve-related therapies. Additionally, the study documented qualitative features, such as the nerve’s superficial trajectory and branching patterns. Given its pilot nature, the findings of this study will contribute to a broader framework for future comprehensive investigations, ultimately enhancing the precision of clinical and surgical applications related to the sural nerve in the Lithuanian population.

### 2.2. Population and Ethics

The study was conducted at the Department of Anatomy, Histology, and Anthropology, Vilnius University Faculty of Medicine, Lithuania. Nine formalin-fixed adult cadavers (comprising eight females and one male) were used for dissection. The age of the cadaveric specimens ranged from 37 to 88 years, with a mean age of 72.7 years. All cadavers were adults and all donors have provided written informed consent (using special notaries’ approved forms) to allow for the conduct of scientific research with their bodies after death. The cadavers used in this study were ethically obtained through body donation programs, following all legal and ethical guidelines.

### 2.3. Anatomical Measurements and Dissection

This study used precise and careful dissection techniques to collect data on sural nerve variations. Standard dissection instruments were employed, and measurements were recorded using a measuring tape and an RS PTO Digital Caliper with an accuracy of 0.01 mm. Each measurement was taken five times by the same researcher, and the final values were calculated as the mean of these measurements.

The dissection process began with a horizontal incision at the junction of the middle and lower third of the thigh and another at the inferior end of the lateral malleolus (LM). These two horizontal incisions were connected by a vertical incision. The skin was carefully reflected bilaterally, exposing the underlying structures.

The superficial fascia in the lower third of the leg was exposed, allowing the identification of the sural nerve in conjunction with the small saphenous vein (SSV). The sural nerve was traced proximally to examine its formation point and the site where it penetrated the deep fascia. The point of penetration was measured from a bony landmark, specifically the head of the fibula.

Following the exposure of the deep fascia, the medial sural cutaneous nerve was traced between the two heads of the gastrocnemius muscle to its origin from the tibial nerve in the popliteal fossa. The peroneal communicating branch was traced proximally from its junction with the medial sural cutaneous nerve to its origin, either from the lateral sural cutaneous nerve or directly from the common peroneal nerve trunk. The origins of all components of the sural nerve complex were documented.

**Figure 1:**
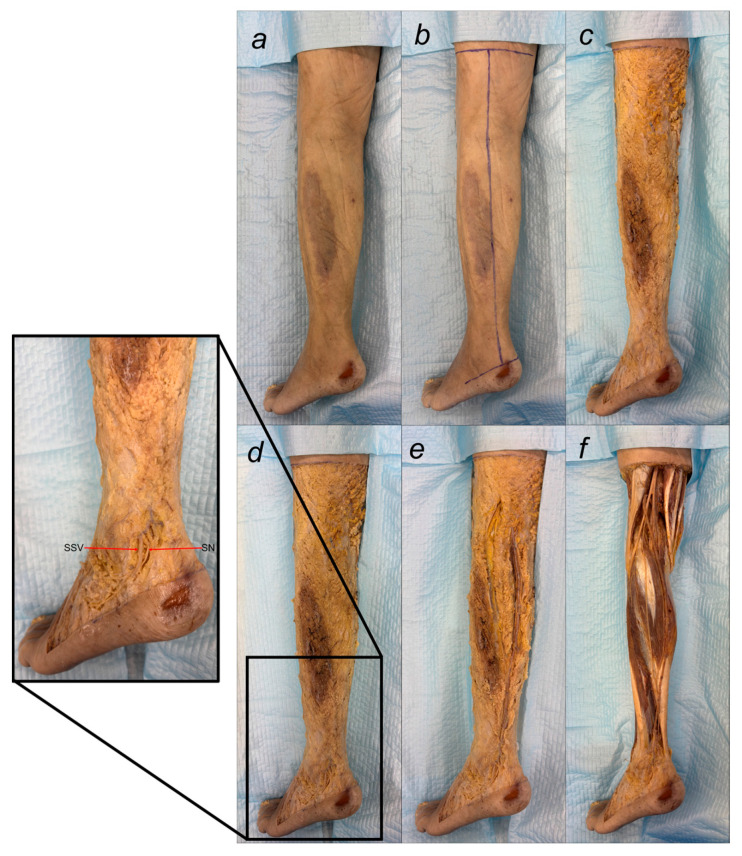
Step-by-step dissection of the sural nerve in the lower limb. (**a**) Intact leg prior to dissection. (**b**) Incisions marked for dissection. (**c**) Skin reflected, exposing the fat layer. (**d**) The small saphenous vein (SSV) was identified; after removing the surrounding adipose tissue, the sural nerve (SN) of the calf was exposed. (**e**) Nerve traced proximally to its origin: tibial and common peroneal nerves. (**f**) Fat removed, showing the complete nerve course and its variations.

After removing the surrounding adipose tissue, detailed observations of the sural nerve were recorded. Each component of the nerve complex, including its origin and course, was documented. Dissection measurements included leg length, sural nerve length, and the length of the sural communicating branch. The distance from the deep fascia penetration and sural nerve formation point to the head of the fibula was measured, as was the total SN length from its formation to the posterior border of the lateral malleolus. The proximal sural nerve diameter was measured at the union site. Additionally, the distances from the posterior border and distal tip of the lateral malleolus were recorded. Furthermore, the diameters and lengths of the tibial nerve, common peroneal nerve, medial sural cutaneous nerve, lateral sural cutaneous nerve, and peroneal communicating nerve were recorded. Finally, the symmetry of sural nerve formation was evaluated (Figure 1).

### 2.4. Classification of Sural Nerve Formation Variations

**Figure 2:**
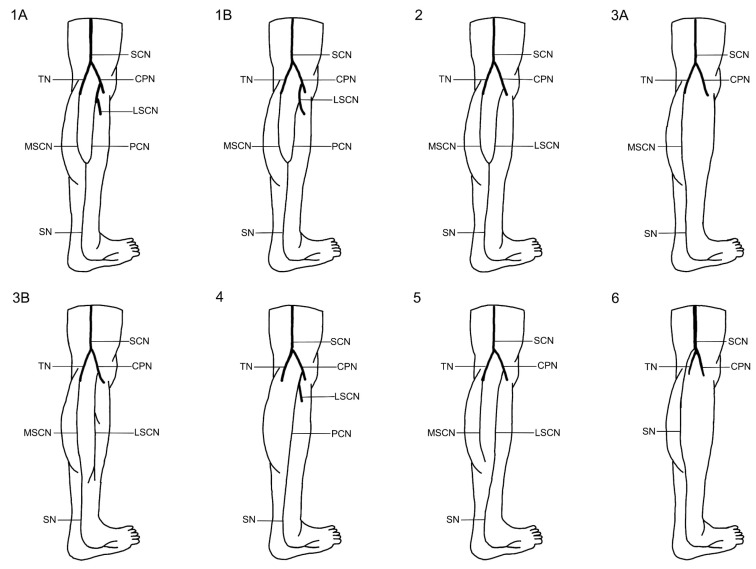
Classification of variations in the formation of the sural nerve by P.K. Ramakrishnan et al. SN—sural nerve; SCN—sciatic nerve; TN—tibial nerve; CPN—common peroneal nerve; MSCN—medial sural cutaneous nerve; LSCN—lateral sural cutaneous nerve; PCN—peroneal communicating nerve. Image Credits: Artur Airapetian.

An integrated classification system for sural nerve formation variations has evolved based on a thorough review of the literature and the standardized framework presented by P.K. Ramakrishnan et al. [4], categorizing SN formation into six main types to elucidate the contributions from the medial sural cutaneous nerve, lateral sural cutaneous nerve, and peroneal communicating nerve. In this system, Type 1 is subdivided into 1A and 1B, with Type 1A defined as the formation of the SN by the union of the MSCN of the tibial nerve and the PCN arising from the common peroneal nerve, and Type 1B characterized by the union of the MSCN of the TN with the PCN arising directly from the LSCN of the CPN. Type 2 involves the union of the MSCN of the TN and the LSCN of the CPN, while Type 3 is further divided into 3A and 3B; Type 3A is marked by the continuation of the MSCN with both the PCN and LSCN absent, and Type 3B by the MSCN continuing alongside an independently present LSCN with an absent PCN. Additionally, Type 4 describes an SN formed solely by the PCN, Type 5 involves an SN formed solely by the LSCN—with the MSCN being either independent or absent—and Type 6 represents an SN that arises directly from the sciatic nerve (SCN). This detailed classification provides a robust framework for understanding the anatomical variations in SN formation and serves as a critical basis for the assessment of these variations in the present study (Figure 2).

### 2.5. Statistical Analysis

Descriptive and analytical statistical methods were used to analyze the data. Pearson’s χ^2^ test was used to detect differences in distribution across limb sides and Fisher’s exact test was used to detect differences in the expected frequencies of less than 5 frequencies per cell. Mann–Whitney and Student’s *t*-tests were used to make a comparative analysis between sides and formation types. The difference was considered statistically significant for *p* ≤ 0.05. Data analysis was carried out using SPSS version 26.0 (IBM Corp, Armonk, NY, USA) and Rstudio version 4.2.2 (PBC Corp., Boston, MA, USA).

## 3. Results

### 3.1. Variations in Sural Nerve Formation in Cadaveric Specimens

The meta-analysis conducted by P.K. Ramakrishnan et al. systematically reviewed a vast body of literature and refined the classification of sural nerve formation variations [4]. However, these formation types were not previously demonstrated in cadaveric specimens.

**Figure 3:**
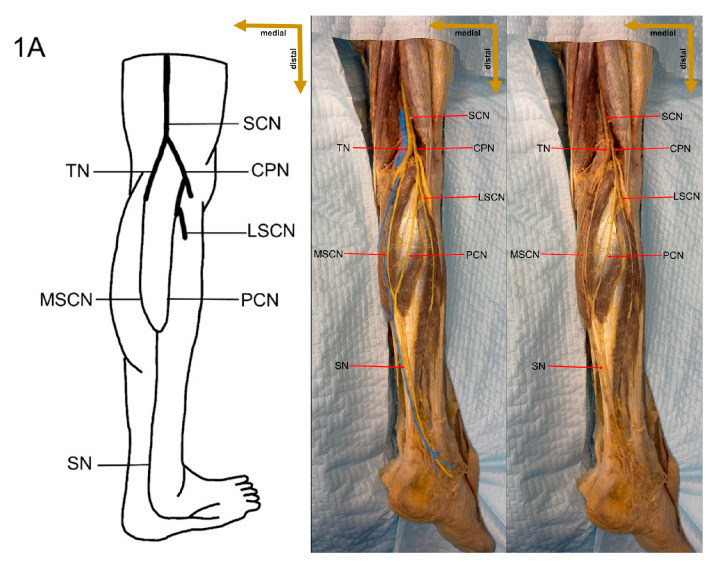
Type 1A formation of sural nerve.

**Figure 4:**
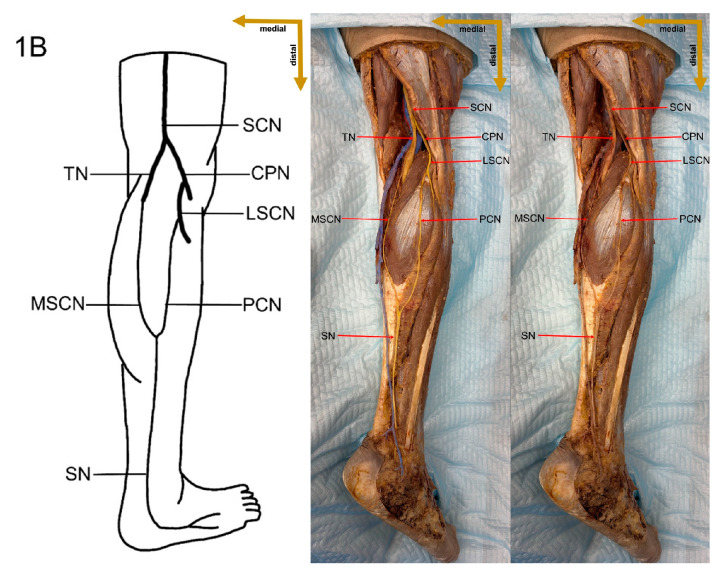
Type 1B formation of sural nerve.

**Figure 5:**
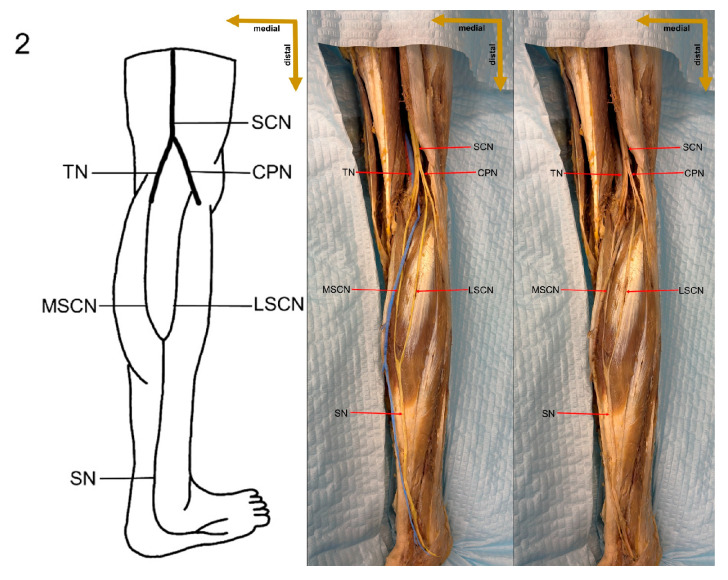
Type 2 formation of sural nerve.

**Figure 6:**
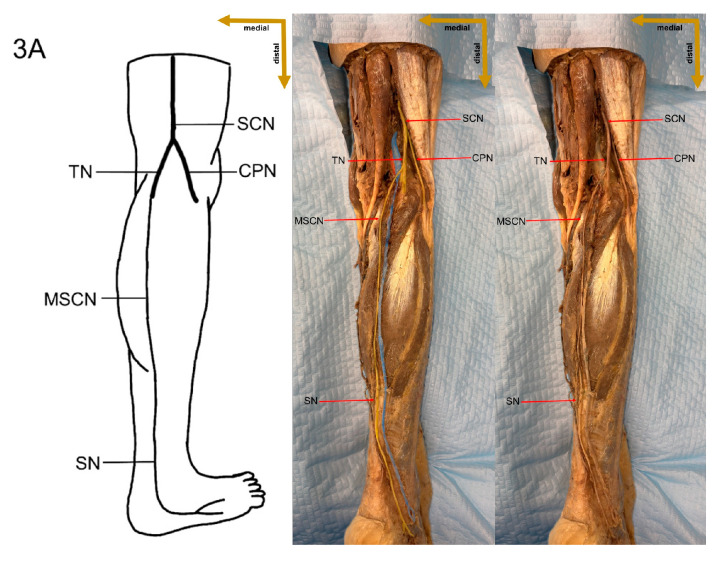
Type 3A formation of sural nerve.

**Figure 7:**
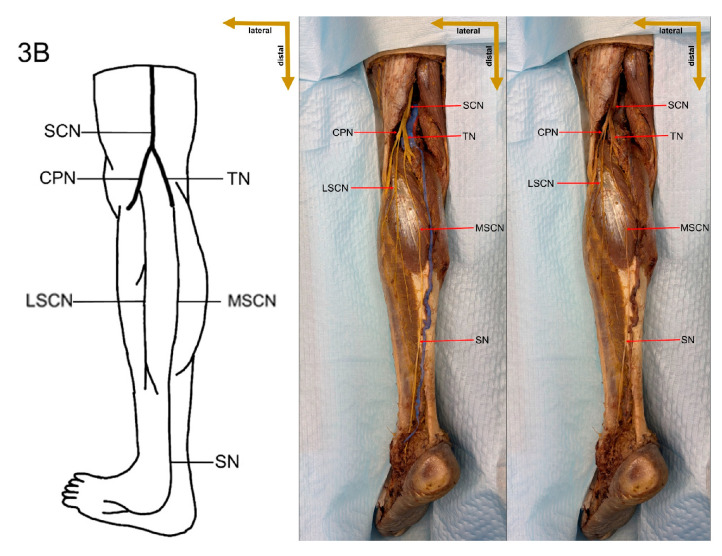
Type 3B formation of sural nerve.

**Figure 8:**
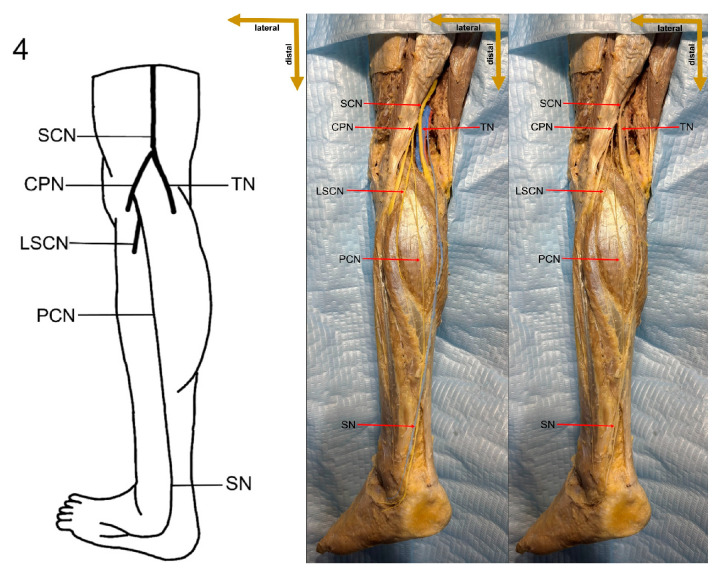
Type 4 formation of sural nerve.

**Figure 9:**
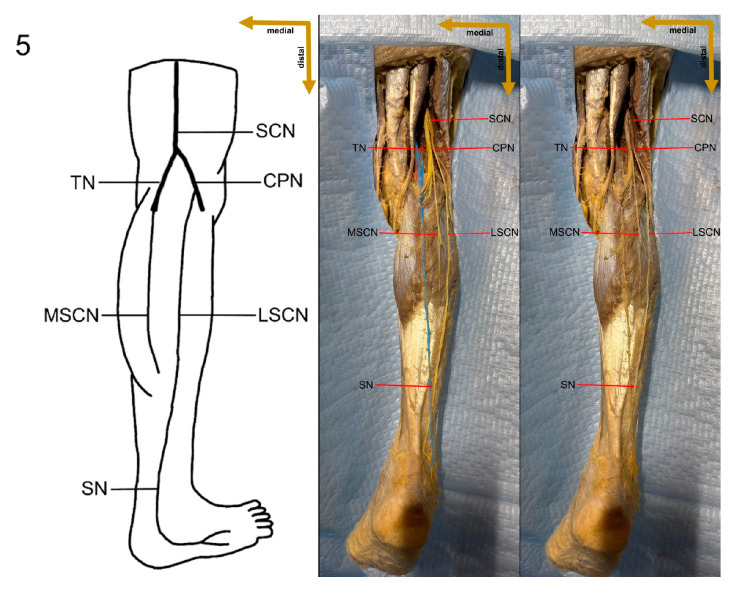
Type 5 formation of sural nerve.

In the present study, a detailed cadaveric dissection was performed to provide direct anatomical validation of the sural nerve formation variations. This approach ensures a precise and tangible representation of the sural nerve’s morphological diversity, bridging the gap between theoretical classifications and actual anatomical findings. The identified SN formation types are illustrated in Figure 3, Figure 4, Figure 5, Figure 6, Figure 7, Figure 8 and Figure 9, offering a comprehensive reference for anatomical and clinical applications.

### 3.2. Sural Nerve Formation Characteristics

The distribution of sural nerve formation variations by side, formation type, and site of union in our study is summarized in Table 1. Type 1A formation was present in 4 of 18 limbs (22.2%), with an equal distribution between the left (2 of 9 limbs, 22.2%) and right (2 of 9 limbs, 22.2%) sides. Type 1B was observed exclusively on the left side in 1 of 9 limbs (11.1%), representing 5.6% of total cases. Type 2 was noted in 3 of 18 limbs (16.7%), with 2 of 9 limbs (22.2%) on the left and 1 of 9 limbs (11.1%) on the right. Type 3A was the most common, occurring in 6 of 18 limbs (33.3%), with an equal split between the left (3 of 9 limbs, 33.3%) and right (3 of 9 limbs, 33.3%) legs. Type 3B formation was identified only on the right side in 2 of 9 limbs (22.2%), accounting for 11.1% of the overall cases. Additionally, SN formation Type 4 was observed in 1 of 18 limbs (5.6%) on the right side, and formation Type 5 was seen in 1 of 18 limbs (5.6%) on the left side; no instances of Type 6 were recorded. Statistical analysis comparing formation type between the sides yielded a *p*-value of 0.743, indicating no significant difference (Table 1).

The formation type of the SN was categorized into two groups: two-contributor formations (MSCN + PCN or MSCN + LSCN) and single-contributor formations (MSCN, LSCN, or PCN). Two-contributor formations were present in 8 of 18 limbs (44.4%), with 3 of 9 limbs (33.3%) on the left and 5 of 9 limbs (55.6%) on the right. In contrast, single-contributor formations predominated in 10 of 18 limbs (55.6%), with a higher frequency on the left (6 of 9 limbs, 66.7%) compared to the right (4 of 9 limbs, 44.4%) (Table 1).

The site of union of the SN within the leg was further examined. Union in the upper half of the leg occurred in 8 of 18 limbs (44.4%), with 3 of 9 limbs (33.3%) on the left and 5 of 9 limbs (55.6%) on the right. Conversely, union in the lower half of the leg was found in 10 of 18 limbs (55.6%), with 6 of 9 limbs (66.7%) on the left and 4 of 9 limbs (44.4%) on the right. The *p*-value for the site of union was 0.637, suggesting no statistically significant difference between the left and right sides (Table 1).

### 3.3. Characteristics of Sural Nerve and Its Forming Structures

The sural nerve and its contributing branches, such as the lateral sural cutaneous nerve, medial sural cutaneous nerve, and peroneal communicating nerve, are thoroughly evaluated morphometrically in this article. With data shown as range, mean ± standard deviation (SD), and median (Q1–Q3), the study emphasizes both diameter and length. With possible clinical ramifications for surgical and neurophysiological applications, the findings provide insight into anatomical diversity (Table 2).

As a main sensory nerve in the lower limb, the sural nerve has the largest mean diameter (2.48 ± 0.68 mm), with a wide range of 1.72–3.74 mm, according to the diameter measurements. Of the contributing branches, the lateral sural cutaneous nerve had the smallest diameter (1.95 ± 0.24 mm) and the peroneal communicating nerve had the second-largest diameter (2.11 ± 0.49 mm), followed by the medial sural cutaneous nerve (2.06 ± 0.19 mm). In contrast to the more constant diameters of the MSCN and LSCN, the sural and peroneal connecting nerves have a comparatively higher standard deviation, indicating greater anatomical variability (Table 2).

The longest nerve in terms of length was the sural nerve (21.99 ± 6.27 cm, range: 10.94–37.46 cm), which was closely followed by the peroneal communicating nerve (21.36 ± 9.66 cm, range: 6.23–32.44 cm) and the medial sural cutaneous nerve (22.14 ± 6.72 cm, range: 7.93–35.39 cm). The shortest nerve was the lateral sural cutaneous nerve (19.51 ± 7.60 cm, range: 6.54–30.96 cm). The large range of values, particularly in the PCN and MSCN, emphasizes the significant variation in these nerves’ length and course, which may be caused by individual variations in their development and merging patterns (Table 2).

Table 3 provides a comparative evaluation of the diameter and length of the sural nerve and its contributing branches, analyzing potential differences between the left and right lower limbs. The statistical analysis includes mean values, *t*-values, and *p*-values, with significance levels set to determine whether variations exist between sides.

The sural nerve shows a larger mean diameter on the right leg (2.64 mm) compared to the left leg (2.03 mm); however, the *p*-value (0.453) indicates no statistical significance. Among its contributing branches, the medial sural cutaneous nerve demonstrated a slightly larger diameter on the right leg (2.10 mm) than the left leg (2.00 mm), but this difference was also not statistically significant (*p* = 0.272). Similarly, the lateral sural cutaneous nerve showed a larger diameter on the right leg (1.99 mm) compared to the left (1.92 mm), with *p* = 0.626. Conversely, the peroneal communicating nerve (PCN) exhibited a slight low diameter on the right leg (2.03 mm) compared to the left (2.19 mm) (*p* = 0.740). These preliminary findings suggest that slight variations exist between the left and right legs; however, none of the differences in nerve diameter reached statistical significance, likely due to the small sample size. Therefore, no definitive conclusions can be drawn, and further studies with larger samples are needed to confirm these observations (Table 3).

In terms of nerve length, the sural nerve was longer in the right leg (23.61 cm) compared to the left leg (20.38 cm); however, the difference did not reach statistical significance (*p* = 0.287). The lateral sural cutaneous nerve exhibited almost identical mean lengths between the right (19.69 cm) and left (19.33 cm) legs (*p* = 0.939). The medial sural cutaneous nerve was slightly shorter on the right leg (20.97 cm) compared to the left (23.45 cm) (*p* = 0.467). Similarly, the peroneal communicating nerve showed a slight shorter in length on the right side (19.68 cm) compared to the left (23.03 cm) (*p* = 0.718) (Table 3).

The data reveal a statistically significant difference in sural nerve diameter between the two formation types (*p* = 0.001). The SN was considerably thicker in the two-contributor formation group (3.17 mm) compared to the single-contributor formation group (1.93 mm). This difference is substantial and suggests that measuring the diameter of the sural nerve could be used as a predictor of its formation type. Greater sural nerve diameter could be associated with SN formation Types 1 and 2, while lower diameter with Types 3, 4, 5, and 6 (Table 4).

By contrast, the contributing branches—the lateral sural cutaneous nerve (1.85 mm vs. 2.00 mm, *p* = 0.933) and the medial sural cutaneous nerve (1.97 mm vs. 1.93 mm, *p* = 0.809)—did not exhibit significant differences in diameter between formation types. This suggests that, while the individual nerves retain relatively stable diameters regardless of their final anatomical configuration, the fusion of two branches significantly increases the overall thickness of the sural nerve (Table 4).

Regarding nerve length, a similar trend was observed. The sural nerve was significantly longer in the two-contributor formation group (25.80 cm) compared to the single-contributor group (18.96 cm, *p* = 0.016). This suggests that the fusion of two branches contributes not only to increased diameter but also to an extended nerve trajectory (Table 4).

The medial cutaneous nerve (24.55 cm vs. 19.43 cm, *p* = 0.119) and lateral cutaneous nerve (20.38 cm vs. 19.08 cm, *p* = 0.795) showed minor length variations between formation types, though these differences were not statistically significant. The lack of significance in the length of the contributing nerves suggests that variation in sural nerve formation does not originate from differences in the lengths of its parent branches but rather from their fusion and convergence patterns (Table 4).

The results indicate significant differences in the diameter of the medial sural cutaneous nerve and peroneal communicating nerve, depending on the site of sural nerve formation. The MSCN was significantly larger when the SN formed in the upper half of the leg (2.15 mm vs. 1.97 mm, *p* = 0.043), while the PCN also exhibited a significantly greater diameter in upper-half formations (2.48 mm vs. 1.74 mm, *p* = 0.049). No significant differences were observed in the lateral sural cutaneous nerve (*p* = 0.755) or the sural nerve itself (*p* = 0.307) (Table 5).

While the reason for these diameter differences remains unclear, it may be related to the degree of contribution from each nerve or variability in neural convergence patterns. Further studies may be needed to clarify these observations.

A clear and statistically significant difference in nerve length was observed, aligning with anatomical expectations. The MSCN and PCN were significantly longer when the SN formed in the lower half of the leg (*p* = 0.009 and *p* = 0.039, respectively). This finding suggests that, when these nerves are longer, their convergence point is lower, leading to a shorter sural nerve (18.42 cm) compared to formations in the upper half (26.48 cm, *p* = 0.003) (Table 5).

This study evaluates the distribution of symmetrical and asymmetrical variations in sural nerve formation across different individuals. The data indicate that a symmetrical formation of the sural nerve was observed in 55.6% of cases (5 out of 9), while asymmetrical formation was present in 44.4% of cases (4 out of 9) (Table 6).

**Figure 10:**
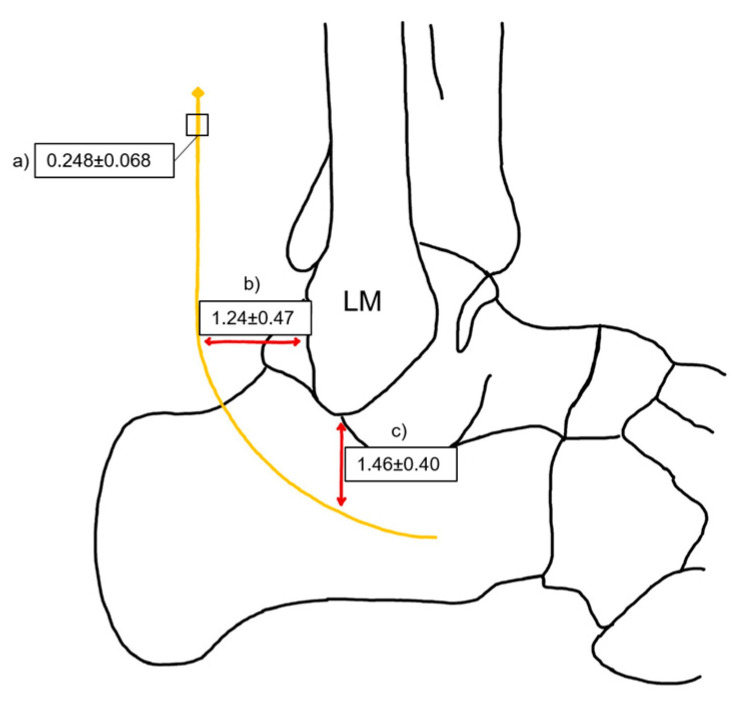
Relationship between the sural nerve (yellow line) and the lateral malleolus (LM). (**a**) The mean diameter of SN. (**b**) Mean distance of the posterolateral aspect of LM to the SN. (**c**) Mean distance from inferior apex of LM to the SN. Image credits: Artur Airapetian.

The anatomical relationship between the sural nerve and the lateral malleolus was quantitatively assessed in both horizontal and vertical orientations. The horizontal measurement represents the distance from the posterolateral aspect of the lateral malleolus to the sural nerve, while the vertical measurement refers to the distance from the inferior apex of the lateral malleolus to the sural nerve. The mean diameter of SN is 0.248 cm with SD 0.068 cm. The horizontal distance from the posterior LM to the SN had a mean of 1.24 cm with a standard deviation of 0.47 cm. The vertical distance from the inferior apex of the LM to the SN had a mean of 1.46 cm, with an SD of 0.40 cm (Figure 10).

## 4. Discussion

The sural nerve shows significant anatomical variation across populations, as demonstrated by our study and previous research. In our sample of 18 limbs from Lithuania, Type 1 formation was observed in 5 limbs (27.8%), which is considerably lower precentage than that reported by Huelke (284 of 352 limbs, 80.7%) [5] and is lower precentage than findings from Serbia (117 of 200 limbs, 58.5%) [6] and India (36 of 50 limbs, 72%) [8], but similar precentage to Shankar et al. (30 of 102 limbs, 29.4%) [7]. Type 2 was identified in 3 limbs (16.6%), a rate lower precentage than that observed in Thailand (102 of 152 limbs, 67.1%) [10] and Korea (20 of 26 limbs, 76.9%) [34], yet comparable to reports from Serbia (18 of 200 limbs, 9%) [6] and the USA (18 of 208 limbs, 8.7%) [9]. Notably, Type 3 was found in 8 limbs (44.4%), which exceeds the precentage frequencies reported in Serbia (52 of 200 limbs, 26%) [6], India (27 of 102 limbs, 26.5%) [7], and the USA (72 of 208 limbs, 34.6%) [9]. Additionally, Type 4 was present in 1 limb (5.6%), a higher precentage rate than that documented by Huelke (1 of 352 limbs, 0.3%) [5] and Steele et al. (1 of 208 limbs, 0.5%) [9] but lower precentage than the 22.5% observed by Shankar et al. (23 of 102 limbs) [7]. Type 5 formation was also seen in 1 limb (5.6%), which is higher precentage than the rates from Serbia (3 of 200 limbs, 1.5%) [6] and the USA (1 of 208 limbs, 0.5%) [9], while Type 6 was absent, consistent with several studies, although Shankar et al. reported it in 14 of 102 limbs (13.7%) [7]. Types 7 and 8 were not observed, aligning with the findings of Huelke [5] and Kavyashree et al. [8], whereas Steele et al. [9] documented these types in 30 of 208 limbs (14.4%) and Pyun and Kwon documented them in 2 of 26 limbs (7.7%) [34]. These inter-study differences may reflect genetic SN formation variations between populations, as genetic predispositions can influence peripheral nerve morphology (Table 7) [7,10].

Analysis of the site of sural nerve formation across the leg reveals notable inter-study differences that may reflect population-based anatomical variability. In our study, SN formation in the upper quarter of the leg was observed in 1 of 18 limbs (5.6%), which is slightly higher precentage than that reported by Ugrenovic et al. [6] (3 of 200 limbs, 1.6%) and Kavyashree et al. [8] (1 of 50 limbs, 2.8%), yet much lower precentage than that documented by Huelke (86 of 352 limbs, 24.3%) [5]. In the second quarter of the leg, our findings showed SN formation in 7 of 18 limbs (38.8%), a frequency higher precentage than that noted by Huelke [5] (59 of 352 limbs, 16.9%) and Ugrenovic et al. [6] (56 of 200 limbs, 28.0%), and considerably higher precentage than Kavyashree et al. (3 of 50 limbs, 5.6%) [8]. In the third quarter, which encompasses the popliteal fossa and proximal calf region, our study reported SN formation in 9 of 18 limbs (50.0%); this aligns with the findings of Ugrenovic et al. [6] (130 of 200 limbs, 64.8%) and P’an MT [36] (147 of 286 limbs, 51.5%), while exceeding Huelke’s [5] report (129 of 352 limbs, 36.6%) and Kavyashree et al.’s observation (17 of 50 limbs, 33.3%) [8]. Finally, in the fourth quarter of the leg, corresponding to the distal calf and ankle region, our study found SN formation in 1 of 18 limbs (5.6%), a value matching that of Ugrenovic et al. [6] (11 of 200 limbs, 5.6%), but substantially lower precentage than those reported by Kavyashree et al. [8] (29 of 50 limbs, 58.3%) and Huelke (78 of 352 limbs, 22.2%) (Table 8) [5].

Table 9 compares the symmetry of sural nerve formation between legs across different populations. In the USA, Huelke (1957) reported symmetry in 291 of 352 cases (82.7%) and asymmetry in 61 of 352 cases (17.3%) [5]. In Serbia, Urgenovic et al. (2005) found symmetry in 124 of 200 cases (62%) and asymmetry in 76 of 200 cases (38%) [6]. In India, Shankar et al. (2010) observed symmetry in 62 of 102 cases (60.8%) and asymmetry in 40 of 102 cases (39.2%) [7], while Kavyashree et al. (2013) documented symmetry in 30 of 50 cases (60%) and asymmetry in 20 of 50 cases (40%) [8]. In Thailand, Mahakkanukrauh and Chomsung (2002) reported symmetry in 30 of 152 cases (19.7%) and asymmetry in 122 of 152 cases (80.3%) [10]. In China, P’an MT (1939) noted symmetry in 240 of 286 cases (83.9%) and asymmetry in 46 of 286 cases (16.1%) [36]. In our present study in Lithuania, symmetry was observed in 10 of 18 cases (55.6%) and asymmetry in 8 of 18 cases (44.4%). These findings indicate that our Lithuanian sample shows a relatively high prevalence of asymmetry compared to most previous studies—except for the Thailand study [10].

Table 10 shows a comprehensive comparison of sural nerve length across different geographical regions based on various cadaveric studies. Steele et al. reported the longest SN length in the USA (32.97 ± 14.12 cm), suggesting notable anatomical variation among populations [9]. In contrast, Sekiya and Kumaki in Japan recorded the shortest SN length (12.4 ± 6.06 cm) [37]. Intermediate values were observed in studies from India and Thailand, with Kavyashree et al. documenting a mean SN length of 19.02 ± 7.66 cm in India, and Mahakkanukrauh and Chomsung reporting 14.41 ± 5.79 cm in Thailand [8,10]. In our present study in Lithuania, the mean SN length was 21.99 ± 6.27 cm, a value that is closer to the Indian dataset but still longer than those reported in Japan and Thailand (Table 10).

The mean sural nerve diameter and standard deviation across different populations based on various cadaveric studies is shown in Table 11.

The highest mean SN diameter was observed in a study conducted in Thailand by Mahakkanukrauh and Chomsung (2002) (3.61 ± 0.07 mm), indicating relatively low variability in SN thickness [10]. Steele et al. (2021) documented a mean SN diameter of 2.74 ± 0.93 mm in a USA-based study, demonstrating a wider range of individual variation (Table 11) [9].

The present study, conducted in Lithuania, reported an intermediate mean SN diameter of 2.48 ± 0.68 mm. This finding suggests that the SN morphology in the Lithuanian population is closer to that observed in the USA, but smaller than that documented in the Thailand population (Table 11) [9,10].

The distance between the sural nerve and key anatomical landmarks of the lateral malleolus across different populations is shown in Table 12. Notably, the distance from the SN to the posterior border of the LM varies slightly, with the highest mean value recorded in the USA (1.7 ± 0.7 cm) and the lowest in Turkey and Lithuania (1.3 cm) [9,35]. Similarly, the distance from the SN to the distal tip of the LM shows regional differences, with England reporting the highest mean (2.3 ± 0.2 cm), while Lithuania and Turkey present the shortest values (1.5 ± 0.4 cm and 1.3 ± 0.7 cm, respectively) (Table 12) [38].

Understanding these variations in the sural nerve formation is crucial for several reasons. First, the SN plays a vital role in the diagnosis and management of diabetic peripheral neuropathy (DPN). It is a common complication of diabetes, often involving the SN and associated with sensory deficits, neuropathic pain, and an increased risk of non-traumatic lower limb amputations [39,40]. While nerve conduction studies remain the gold standard for DPN diagnosis, imaging modalities such as high-frequency ultrasound and SN biopsy offer valuable insights into the morphological changes that occur in neuropathic conditions [41,42]. The early and accurate identification of these changes is critical for timely intervention and improved patient outcomes. Given the clinical significance of the SN, it is essential to perform tissue biopsies when necessary and to account for potential variations in its length, diameter, and localization across different populations. These anatomical differences may influence biopsy accuracy, nerve conduction studies, and surgical interventions, emphasizing the need for population-specific reference data. A comprehensive understanding of these parameters enhances diagnostic precision and ensures more effective treatment strategies, particularly in diabetic patients who are at high risk for neuropathic complications [43,44].

Furthermore, isolated SN neuropathy (mononeuropathy) and SN entrapment are clinical entities that underscore the importance of detailed anatomical knowledge. The SN’s superficial location makes it particularly vulnerable to trauma, compression from space-occupying lesions, and iatrogenic injury during surgical procedures [45,46]. In cases of mononeuropathy, patients may present with pain, numbness, and paresthesia, which can mimic other neurological conditions [47]. Sural nerve entrapment similarly manifests with pain, burning, tenderness, and abnormal sensations in the posterolateral region of the distal leg and the lateral aspect of the foot extending to the fifth digit [48]. Precise anatomical mapping, therefore, facilitates correct diagnosis and targeted treatment.

The sural nerve is widely utilized in nerve grafting due to its favorable anatomical properties, making it a preferred donor nerve in reconstructive surgeries. Its straight course, minimal branching, and optimal caliber contribute to successful nerve regeneration [49]. Sural nerve grafting is particularly valuable for repairing long nerve defects and is often chosen over other autologous grafts for its specific advantages [50]. Nerve grafts are typically required for segmental nerve loss exceeding 1–2 cm, as a careful mobilization of nerve stumps can often reduce smaller gaps. Synthetic conduits, however, generally provide reliable results only for defects smaller than 5 mm [51]. The success of nerve grafting or direct nerve repair depends on the presence of viable proximal and distal nerve stumps. When a proximal stump is unavailable, such as in skull-base injuries, a nerve transfer to the distal stump may provide a more effective reconstructive approach [52,53]. The considerable length of the sural nerve makes it particularly valuable for facial nerve reinnervation, allowing cross-face grafting from a healthy facial nerve to the paralyzed side [54,55]. Additionally, sural nerve grafts are frequently used in nerve elongation procedures, particularly in cases of brachial plexus injuries [56].

The effectiveness of sural nerve grafting is influenced by several anatomical factors, including nerve length, diameter, and branching pattern. Our study highlights significant variability in SN morphology across different populations, with notable differences in both length and diameter. These parameters are crucial in reconstructive surgery, as the harvested nerve must be appropriately matched to the defect to ensure optimal functional recovery. In our study, the mean sural nerve length (21.99 ± 6.27 cm) falls within an intermediate range compared to previous reports, suggesting that graft suitability may differ among populations. Likewise, the diameter of the SN, which averaged 2.48 ± 0.68 mm in our sample, plays a key role in graft integration and revascularization. A thicker nerve may provide a more robust scaffold for axonal regeneration, while a thinner nerve may be more prone to atrophy or incomplete reinnervation [57]. These findings underscore the importance of considering population-specific anatomical variations when selecting donor nerves for grafting procedures.

Beyond limb and facial nerve repair, sural nerve grafts have also been used in corneal neurotization, particularly in patients with neurotrophic keratitis—a condition characterized by corneal anesthesia, a loss of the blink reflex, and reduced tear production, leading to ulceration, scarring, and eventual corneal opacification. In such cases, sural nerve grafts, along with the great auricular nerve, have been used to restore corneal sensation by connecting a functional sensory nerve, such as the supratrochlear or supraorbital nerve, to the affected cornea. This enables axonal regeneration and sensory restoration [58]. Given the variability in SN morphology observed in our study, the success of such procedures may be influenced by regional differences in nerve structure. For instance, variations in SN diameter could affect the density of regenerating axons and the overall sensory recovery in corneal neurotization [59]. These diverse applications highlight the clinical significance of the sural nerve and emphasize the necessity of understanding its anatomical variations to improve surgical planning and patient outcomes.

### The Limits of the Study

One of the primary limitations of this study is the small sample size. A total of nine cadavers (18 limbs) were examined, which restricts the generalizability of the findings to the broader Lithuanian population and other ethnic groups. Comparative studies in the literature typically include a minimum of 25 cadavers, providing a higher level of evidence and greater statistical power. Although notable differences in sural nerve formation were observed within the Lithuanian population, the limited sample size reduces the reliability of these findings and precludes definitive conclusions. Future studies with larger and more diverse samples are necessary to validate these results and enhance their applicability.

Another limitation of this study is the gender imbalance in the sample, which included eight female cadavers and only one male cadaver. This uneven distribution prevents a meaningful analysis of sex differences in sural nerve morphology. While a direct comparison was possible, interpreting the results in this way would be inappropriate. Instead, the study presents morphological data that future researchers can use for comparative analysis. To better understand potential gender-related differences in sural nerve formation, future studies should include a more balanced sample.

Despite these limitations, this study provides valuable preliminary data on sural nerve morphology in the Lithuanian population. This study is among the first to clearly define its methodology, providing both a detailed explanation and visual representation through images (Figure 1). These images illustrate each step of the process, enhancing clarity and reproducibility. In addition to the theoretical description of sural nerve variations, the study presents these variations directly on cadaveric specimens. This visual approach allows future researchers to better understand the structures and how to identify them, improving accuracy in anatomical studies.

## 5. Conclusions

In the Lithuanian population, the most common sural nerve formation variant was Type 3, which was observed in 8 out of 18 limbs (44.4%). This study also evaluates the distribution of symmetrical and asymmetrical variations in the formation of the sural nerve across different individuals. The results indicate that a symmetrical formation of the sural nerve was found in 55.6% of cases (*n* = 5), while asymmetrical formation was present in 44.4% of cases (*n* = 4). The most frequent anatomical locations of the sural nerve were identified in the second (7 out of 18 limbs, 38.8%) and third (9 out of 18 limbs, 50%) quarters of the lower leg. Additionally, a statistically significant difference (*p* = 0.001) was observed in the diameter of the sural nerve between anatomical variants where the nerve arises from the fusion of two components (3.17 mm) as opposed to those in which the sural nerve continues from a single origin (1.93 mm.). This finding suggests a potential correlation between sural nerve thickness and its morphological classification. Specifically, thicker sural nerves are more commonly associated with Type 1 and Type 2 formations, while thinner sural nerves tend to be observed in Type 3, Type 4, Type 5, and Type 6 formations. Further research is needed to explore the clinical and anatomical implications of these variations. Furthermore, in our study, the mean length of the sural nerve was found to be 21.99 ± 6.27 cm, with an average diameter of 2.48 ± 0.68 mm. Additionally, the mean distance from the sural nerve to the posterior border of the lateral malleolus was 1.3 ± 0.5 cm, and the mean distance from the sural nerve to the distal tip of the lateral malleolus was 1.5 ± 0.4 cm. These findings provide valuable insights into the anatomical characteristics of the sural nerve within the Lithuanian population and highlight the importance of further investigation into the clinical relevance of these variations.

## Figures and Tables

**Table 1:** Distribution of sural nerve variations, formation type, and site of union by lower limb side.

Variation	Side		Total (%)	*p*-Value
	Left Leg (%)	Right Leg (%)		
1A	2 (22.2%)	2 (22.2%)	4 (22.2%)	0.743
1B	1 (11.1%)	0 (0.0%)	1 (5.6%)	
2	2 (22.2%)	1 (11.1%)	3 (16.7%)	
3A	3 (33.3%)	3 (33.3%)	6 (33.3%)	
3B	0 (0.0%)	2 (22.2%)	2 (11.1%)	
4	0 (0.0%)	1 (11.1%)	1 (5.6%)	
5	1 (11.1%)	0 (0.0%)	1 (5.6%)	
6	0 (0.0%)	0 (0.0%)	0 (0.0%)	
Formation Type of SN				0.637
Two-Contributor Formation (MSCN + PCN, MSCN + LSCN)	3 (33.3%)	5 (55.6%)	8 (44.44%)	
Single-Contributor Formation (MSCN, LSCN, PCN)	6 (66.7%)	4 (44.4%)	10 (55.55%)	
Site of union of the sural nerve formation in the leg				0.637
Upper half of leg	3 (33.3%)	5 (55.6%)	8 (44.44%)	
Lower half of leg	6 (66.7%)	4 (44.4%)	10 (55.55%)	

**Table 2:** Morphometric analysis of the sural nerve and its contributing branches.

**Nerve**	**Diameter (mm)**		
	**Range**	**Mean ± SD**	**Median (Q1–Q3)**
Lateral sural cutaneous nerve	1.61–2.39	1.95 ± 0.24	1.94 (1.75–2.04)
Medial sural cutaneous nerve	1.66–2.38	2.06 ± 0.19	2.10 (1.93–2.21)
Peroneal communicating nerve	1.64–2.98	2.11 ± 0.49	1.98 (1.78–2.26)
Sural nerve	1.72–3.74	2.48 ± 0.68	2.1 (1.93–3.19)
**Nerve**	**Length (cm)**		
	**Range**	**Mean ± SD**	**Median (Q1–Q3)**
Lateral sural cutaneous nerve	6.54–30.96	19.51 ± 7.60	19.56 (14.45–23.91)
Medial sural cutaneous nerve	7.93–35.39	22.14 ± 6.72	23.15 (18.75–26.37)
Peroneal communicating nerve	6.23–32.44	21.36 ± 9.66	21.38 (17.07–28.64)
Sural nerve	10.94–37.46	21.99 ± 6.27	21.85 (18.29–25.19)

**Table 3:** Comparative analysis of sural nerve and its branches between left and right legs.

Nerve	Diameter (mm)			
	Mean Value		*t*-Value	*p*-Value
Comparisons	Left Leg	Right Leg		
Lateral sural cutaneous nerve	1.92	1.99	0.50	0.626
Medial sural cutaneous nerve	2.00	2.10	1.14	0.272
Peroneal communicating nerve	2.19	2.03	−0.36	0.740
Sural nerve *	2.03	2.64	-	0.453
	**Length (cm)**			
Lateral sural cutaneous nerve	19.33	19.69	0.08	0.939
Medial sural cutaneous nerve	23.45	20.97	−0.75	0.467
Peroneal communicating nerve	23.03	19.68	−0.39	0.718
Sural nerve	20.38	23.61	1.10	0.287

* Statistical analysis was conducted using the Mann–Whitney U test.

**Table 4:** Morphometric comparison of sural nerve and its branches based on formation.

Nerve	Diameter (mm)			
	Mean Value		*t*-Value	*p*-Value
Comparisons	Two-Contributor Formation (MSCN + PCN, MSCN + LSCN)	Single-Contributor Formation (MSCN, LSCN, PCN)		
Lateral cutaneous nerve *	1.85	2.00	-	0.933
Medial cutaneous nerve	1.97	1.93	0.25	0.809
Sural nerve **	3.17	1.93	10.22	0.001 **
	**Length (cm)**			
Lateral cutaneous nerve	19.08	20.38	−0.27	0.795
Medial cutaneous nerve	19.43	24.55	−1.65	0.119
Sural nerve **	25.80	18.96	2.69	0.016 **

* Statistical analysis was conducted using the Mann–Whitney U test. ** Statistically significant.

**Table 5:** Morphometric comparison of sural nerve and its branches based on the site of union.

Nerve	Diameter (mm)			
	Mean Value		*t*-Value	*p*-Value
Site of Union of the Sural Nerve Formation in the Leg	Upper Half of Leg	Lower Half of Leg		
Lateral sural cutaneous nerve *	1.80	1.98	-	0.755
Medial sural cutaneous nerve **	2.15	1.97	−2.21	0.043 **
Peroneal communicating nerve **	2.48	1.74	−2.80	0.049 **
Sural nerve *	2.89	2.06	-	0.307
	**Length (cm)**			
Lateral sural cutaneous nerve	19.42	19.57	0.03338	0.974
Medial sural cutaneous nerve **	17.95	25.86	2.96	0.009 **
Peroneal communicating nerve **	13.99	28.71	3.02	0.039 **
Sural nerve **	26.48	18.42	−3.49	0.003 **

* Statistical analysis was conducted using the Mann–Whitney U test. ** Statistically significant.

**Table 6:** Distribution of symmetry and asymmetry in sural nerve formation.

	Total
Symmetric	5 (55.6%)
Asymmetric	4 (44.4%)

**Table 7:** Comparisons of SN formations variants.

	Region	Number of Cases	Type 1 (%)	Type 2 (%)	Type 3 (%)	Type 4 (%)	Type 5 (%)	Type 6 (%)	Types 7 and 8 (%)
Huelke (1957) [5]	USA	352 (100%)	284 (80.7%)	-	67 (19%)	1 (0.3%)	-	-	-
Urgenovic et al. (2005) [6]	Serbia	200 (100%)	117 (58.5%)	18 (9%)	52 (26%)	-	3 (1.5%)	-	10 (5%)
Shankar et al. (2010) [7]	India	102 (100%)	30 (29.4%)	-	27 (26.5%)	23 (22.5%)	-	14 (13.7%)	8 (7.8%)
Kavyashree et al. (2013) [8]	India	50 (100%)	36 (72%)	-	14 (28%)	-	-	-	-
Steele et al. (2021) [9]	USA	208 (100%)	86 (41.4%)	18 (8.7%)	72 (34.6%)	1 (0.5%)	1 (0.5%)	-	30 (14.4%)
Mahakkanukrauh and Chomsung (2002) [10]	Thailand	152 (100%)	1 (0.7%)	102 (67.1%)	49 (32.2%)	-	-	-	-
Pyun and Kwon (2008) [34]	Korea	26 (100%)	-	20 (76.9%)	4 (15.4%)	-	-	-	2 (7.7%)
Aktan Ikiz et al. (2005) [35]	Turkey	30 (100%)	18 (60.0%)	3 (10.0%)	5 (16.7%)	2 (6.7%)	2 (6.7%)	-	-
Present study	Lithuania	18 (100%)	5 (27.8%)	3 (16.6%)	8 (44.4%)	1 (5.6%)	1 (5.6%)	-	-

**Table 8:** Comparisons of the site of SN formation.

	Region	Number of Cases	Upper Quarter of the Leg (%)	Second Quarter of the Leg (%)	Third Quarter of the Leg (%)	Fourth Quarter of the Leg (%)
Huelke (1957) [5]	USA	352 (100%)	86 (24.3%)	59 (16.9%)	129 (36.6%)	78 (22.2%)
Ugrenovic et al. (2005) [6]	Serbia	200 (100%)	3 (1.6%)	56 (28.0%)	130 (64.8%)	11 (5.6%)
Kavyashree et al. (2013) [8]	India	50 (100%)	1 (2.8%)	3 (5.6%)	17 (33.3%)	29 (58.3%)
P’an MT (1939) [36]	China	286 (100%)	20 (6.9%)	31 (10.7%)	147 (51.5%)	88 (30.9%)
Present study	Lithuania	18 (100%)	1 (5.6%)	7 (38.8%)	9 (50.0%)	1 (5.6%)

**Table 9:** Symmetry of the formation of the SN between legs.

	Region	Number of Cases	Symmetry (%)	Asymmetry (%)
Huelke (1957) [5]	USA	352 (100%)	291 (82.7%)	61 (17.3%)
Urgenovic et al. (2005) [6]	Serbia	200 (100%)	124 (62%)	76 (38%)
Shankar er al. (2010) [7]	India	102 (100%)	62 (60.8%)	40 (39.2%)
Kavyashree et al. (2013) [8]	India	50 (100%)	30 (60.0%)	20 (40.0%)
Mahakkanukrauh and Chomsung (2002) [10]	Thailand	152 (100%)	30 (19.7%)	122 (80.3%)
P’an MT (1939) [36]	China	286 (100%)	240 (83.9%)	46 (16.1%)
Present study	Lithuania	18 (100%)	10 (55.6%)	8 (44.4%)

**Table 10:** Comparisons of SN length.

	Region	SN Length (Mean) (cm)	SD (cm)
Kavyashree et al. (2013) [8]	India	19.02	7.66
Steele et al. (2021) [9]	USA	32.97	14.12
Mahakkanukrauh and Chomsung (2002) [10]	Thailand	14.41	5.79
Sekiya and Kumaki (2002) [37]	Japan	12.4	6.06
Present study	Lithuania	21.99	6.27

**Table 11:** Comparisons of sural nerve diameters.

	Region	SN Diameter Mean (mm)	SD (mm)
Steele et al. (2021) [9]	USA	2.74	0.93
Mahakkanukrauh and Chomsung (2002) [10]	Thailand	3.61	0.07
Present study	Lithuania	2.48	0.68

**Table 12:** Morphometrics between sural nerve and later malleolus.

	Region	Distance from SN to Posterior Boarder of LM (cm)	Distance from SN to Distal Tip of LM (cm)
Steele et al. (2021) [9]	USA	1.7 ± 0.7	1.8 ± 0.8
Aktan Ikiz et al. (2005) [35]	Turkey	1.3 ± 0.9	1.3 ± 0.7
Solomon et al. (2001) [38]	England	1.4 ± 0.1	2.3 ± 0.2
Present study	Lithuania	1.3 ± 0.5	1.5 ± 0.4

## Data Availability

The datasets analyzed during the current study are available from the corresponding author on reasonable request.
